# Shifts in the Clonal Distribution of Methicillin-Resistant *Staphylococcus aureus* in Kuwait Hospitals: 1992-2010

**DOI:** 10.1371/journal.pone.0162744

**Published:** 2016-09-15

**Authors:** Samar S. Boswihi, Edet E. Udo, Noura Al-Sweih

**Affiliations:** Department of Microbiology, Faculty of Medicine, Kuwait University, Jabriya, Kuwait; Universitatsklinikum Munster, GERMANY

## Abstract

**Background:**

As the epidemiology of methicillin-resistant *Staphylococcus aureus* (MRSA) is constantly changing globally, determining the prevailing MRSA clones in a local healthcare facility is important for better management of infections. This study investigated clonal composition and distribution of MRSA isolates in Kuwait’s hospitals using a combination of molecular typing methods.

**Materials and Methods:**

In total, 400 non-repeat MRSA isolates were obtained between 1992 and 2010 in 13 public hospitals and were characterized using antibiogram, SCC*mec* typing, spa typing, and multilocus-sequence typing. Clonal assignment and detection of virulence factors and antibiotic resistance genes were performed by DNA microarray.

**Results:**

The isolates were resistant to kanamycin (74.2%), erythromycin (69.5%), tetracycline (66.7%), gentamicin (61%), ciprofloxacin, (61%), fusidic acid (53.5%), clindamycin (41.5%), high-level mupirocin resistance (5.2%) and carried *aphA3*, *aacA-aphD*, *ermA*, *ermC*, *mupA*, *tetK*, *tetM*, *fusC* and *far1*. Molecular typing revealed 31 different MRSA clones consisting of ST239-MRSA-III (52.2%), ST22-MRSA-IV (9.2%), ST80-MRSA-IV (7.5%), ST5-MRSA-II/IV/V/VI (6.5%), ST30-MRSA-IV (3.5%), ST241-MRSA-III (2.7%), ST6-MRSA-IV (2.2%), ST36-MRSA-II (2%) and ST772-MRSA-V (1.75%). The isolates differed in the carriage of genes for enterotoxins, Panton–Valentine leukocidin (PVL), toxic shock syndrome toxin (tst-1), arginine catabolic mobile element (ACME) and exfoliative toxins. The number of clones increased from one (ST239-III-t037) in 1992 to 30 in 2010 including ST8-IV-t008 [PVL+] [ACME+] (USA300), ST772-V (Bengal Bay clone) and ST2816 identified for the first time in Kuwait.

**Conclusion:**

The study revealed that the MRSA isolates belonged to diverse clones that changed in numbers and diversity overtime. Although ST239-MRSA-III, a healthcare-associated clone remained the dominant MRSA clone overtime, the newly emerged clones consisted mostly of community-associated.

## Introduction

Methicillin-resistant *Staphylococcus aureus* (MRSA) is a major pathogen causing a wide range of infections [1]. MRSA was initially confined to healthcare systems and were known as healthcare-associated MRSA (HA-MRSA). However, the epidemiology of MRSA changed with the evolution of community-acquired or community-associated MRSA (CA-MRSA) strains among healthy individuals with no previous exposure to healthcare facilities [2]. Characteristically, HA-MRSA are usually multi-resistant and carry SCCmec types I, II or III, while CA-MRSA are usually more susceptible to non-beta-lactam antibiotics and carried SCCmec types IV, V or VI [3].

Molecular typing has provided tools for surveillance and outbreak investigation, which has enhanced the detection of closely related strains [3, 4, 5]. MRSA constitutes a major drug resistance problem in many hospitals and healthcare settings worldwide leading to limited choices of therapeutic options for the treatment of multi-resistant strains [1, 4]. By using epidemiological typing methods such as multilocus-sequence typing (MLST) and SCCmec typing, it is possible to group MRSA strains into different clones [5, 6] and has contributed to the understanding of MRSA transmission in healthcare facilities. The nomenclature of MRSA clones is based on sequence type (ST) and SCCmec types [5]. Most of the epidemic MRSA isolates belong to MLST clonal complexes (CCs) including CC5, CC8, CC22, CC30, and CC45 [5, 6]. Other reported clones include CC6, CC7, CC9, CC12, CC15, CC20, CC59, CC75, CC80, CC88, CC93, CC96/ST154, CC97, CC130, CC121, CC152, CC188, CC361, CC395/ST426, CC398, CC509, CC779 and CC913 [5,6]. Strains belonging to CC97 and CC398 have been associated with livestock (livestock-associated MRSA) [7]. Some MRSA clones are spread globally while others are restricted to specific geographical regions. For example, while ST239-III and ST22-IV are widespread globally, isolates belonging to ST59 and ST93 have limited geographical spread [8, 9, 10, 11]. The ST239-III-MRSA is a well-known pathogen that has been associated with healthcare-associated infections. It is a pandemic clone which has been widely reported from Asia, Europe, Middle East, South and North America [5]. DNA sequence analysis of ST239 isolates obtained from early 1980s by Harris *et al*., [12] has revealed several variants corresponding to Brazilian, Portuguese, Hungarian and Viennese clones. The ST22-IV-MRSA, also known as epidemic MRSA-15, is another healthcare-associated pathogen that has been reported from different parts of the world [5]. ST22-IV-MRSA was initially isolated in the UK in 1991 [13]. Since then it has been reported in other European countries, Australia, Asia, and the Middle East [5, 14, 15]. Using phylogenomic methods to analyze the genome sequence of 193 S. aureus isolates, Holden *et al*., [14] showed that the current pandemic population of EMRSA-15 descends from a healthcare-associated MRSA epidemic that spread throughout England in the 1980s. In recent years, ST22-IV-MRSA has diversified into variants, based on SCC*mec* subtypes, acquisition of PVL and TSST genes, known as UK EMRSA-15/Barnim, UK EMRSA-15/Middle Eastern variant and CC22-MRSA-IV-PVL^+^, variants [5, 15]

The number of MRSA isolates obtained in Kuwait hospitals has increased over the years [16]. Previous studies revealed that MRSA constituted 32% of *S*. *aureus* isolated from clinical samples in Kuwait and consisted of both healthcare-associated and community-associated strains [16, 17]. Despite these studies, there has been no systematic characterization of MRSA isolated in Kuwait hospitals overtime to catalogue changes in their clonal distribution. The purpose of this study was to investigate the number and type of MRSA clones circulating in major Kuwait hospitals and determine their genotypic characteristics. The strains were investigated using a combination of molecular typing techniques including Staphylococcal chromosome cassette *mec* (SCC*mec*) typing, spa typing and multilocus-sequence typing (MLST). Additionally, the study aimed to determine if there were changes in the distribution of the MRSA clones in Kuwait hospitals over time. Tracking the emergence and spread of MRSA and the introduction of new clones will provide information needed to guide policy for appropriate therapy and infection control. In addition, it will provide information about the composition of MRSA clones in Kuwait, which is necessary for epidemiological purposes.

## Materials and Methods

### Collection of MRSA strains

In total 400 non-duplicate MRSA, isolates representing isolates obtained from different clinical (Table 1) samples in 13 government Kuwait hospitals between 1992 and 2010 were investigated. The isolates were selected from a collection of MRSA isolates archived at the MRSA Reference Laboratory in Kuwait. The isolates were previously characterized by pulsed-field gel electrophoresis (PFGE) and interpreted according to the criteria prescribed by Tenover *et al*., [18]. Those selected for this study represented different PFGE patterns obtained in 1992–2010. During the 90’s and early 2000’s, the number of PFGE patterns were small due to the presence of an endemic clone [19, 20] which is reflected in the small numbers of isolates chosen for this study from these years. The distribution of MRSA and numbers of isolates per year is as follows: 1992 (N = 15), 1996 (N = 20), 1997 (N = 32), 1998 (N = 16), 1999 (N = 21), 2001–2002 (39), 2005 (N = 52), 2010 (205).

**Table 1:** Source of MRSA isolates.

Source	No. of isolates (%)
Axilla	8 (2)
Blood	16 (4)
Burn	29 (7.2)
Catheter tip	8 (2)
Endotracheal (ET)	8 (2)
Groin	21 (5.2)
HVS	7 (1.7)
Nasal	92 (23)
*Skin and soft tissue infections	113 (28.2)
Sputum	11 (2.7)
Throat	12 (3)
Tissue	6 (1.5)
Tracheal	9 (2.2)
Umbilical	8 (2)
**Miscellaneous	52 (13)
**Total**	**400**

* Wound (53 samples), Pus (26 samples), bed sore (18 samples), abscess (nine samples), Skin (seven samples).

** MRSA screening swab (11 samples), ear (five samples), Urine (five samples), eye (four samples), catheter tips (four samples), foot swab (three samples), Gastro (three samples), perianal (two samples), fluid (two sample), ulcer (two samples), buttocks (one sample), bronchial lavage (one sample), carbuncle (one sample), central venous pressure (CVP) catheter tip (one sample), femoral vein catheter (FVC) tip (one sample), hairline (one sample), laparoscopy (one sample), percutaneous endoscopic gastrostomy (PEG) site (one sample), peritoneal fluid (one sample), tongue swab (one sample), tracheal secretion (TS) (one sample).

### Antimicrobial susceptibility testing

Antibiotic susceptibility testing was performed by disc diffusion method according to the Clinical Laboratory Standards Institute (CLSI); [21] with the following antimicrobial disks (Oxoid): benzyl penicillin (10U), cefoxitin (30 μg), amikacin (30 μg), kanamycin (30 μg), tobramycin (10 μg), mupirocin (200 μg and 5 μg), gentamicin (10 μg), erythromycin (15 μg), clindamycin (2 μg), Spectinomycin (25 μg), chloramphenicol (30 μg), tetracycline (10 μg), trimethoprim (2.5 μg), fusidic acid (10 μg), rifampicin (5 μg), ciprofloxacin (5 μg), teicoplanin (30 μg), and linezolid (30 μg). Minimum inhibitory concentration (MIC) for cefoxitin, vancomycin and teicoplanin were determined with Etest strips (AB BioMerieux, Marcy l’Etoile, France) according to the manufacturer's instructions. *S*. *aureus* strain ATCC25923 was used as a quality control strain for susceptibility testing. The D-test was used to test for inducible resistance to clindamycin.

Susceptibility to fusidic acid was interpreted according to the British Society to Antimicrobial Chemotherapy (BSAC) [22]. Etest was used to determine the MIC for vancomycin and teicoplanin. The interpretation of the MIC values was based on the antibiotic breakpoint concentration recommended by the CLSI; [21].

### Detection of Panton-Valentine leukocidin (PVL)

PVL was performed for all MRSA isolates using primers and PCR protocol published by Lina *et al*., [23].

### SCC*mec* typing and Staphylococcus protein A (spa) typing

All MRSA isolates were characterized using SCC*mec* typing and spa typing. SCC*mec* typing was performed as described by Zhang *et al*., [24]. Detection of SCC*mec*-IV subtypes IVa, IVb, IVc, IVd, IVg and IVh was determined using multiplex PCR described by Zhang *et al*., [24] and Milheiriço *et al*., [25]. Spa typing was performed as described by Harmsen *et al*., [26].

### Multilocus sequencing typing (MLST)

MLST was performed as described by Enright *et al*., [27] on 103 isolates representing different spa types in 1992–2010. Electronic based upon related sequence types (eBURST) (http://eburst.mlst.net) [28] analysis was performed and assigns each ST that shares at least five of seven identical alleles into a single clonal complex (CC).

### DNA Microarray

DNA microarray was performed for 110 MRSA isolates representing each spa type and sequence type isolated from 1992 to 2010. Genes encoding virulence factors and antibiotic resistance were determined using the ArrayMate Reader DNA Microarray platform with Identibac *S*. *aureus* genotyping Kit 2.0 (Alere Technology, Jena, Germany) following protocols provided by the manufacturer.

## Results

### Detection of genes for PVL among MRSA isolates

The result showed that 62 (15.5%) isolates were positive for PVL, while 338 isolates (84.5%) of the isolates was negative.

### Molecular typing of MRSA isolates

**Fig 1:**
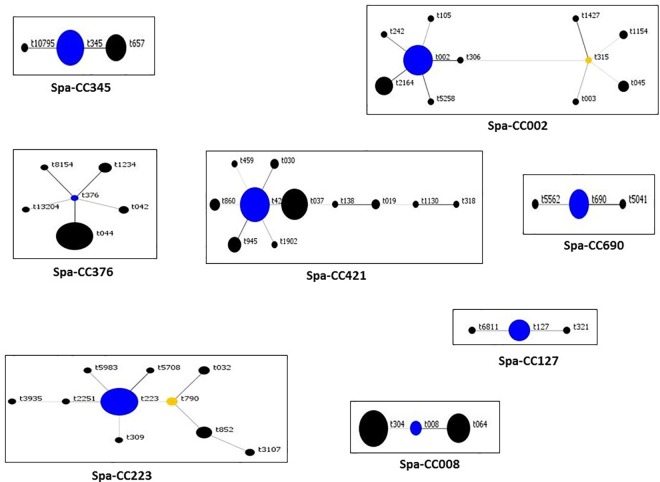
Spa clonal complex (Spa-CC) of MRSA isolates.

The result of SCC*mec* typing revealed five SCC*mec* types including types II (N = 18; 4.5%), III (N = 221; 55.2%), IV (N = 127; 31.7%), V (N = 33; 8.2%), and VI (N = 1; 0.2%). There were 60 spa types with the dominant ones being t421 (N = 86; 21.5%) and t037 (N = 76; 19%). Other common spa types were t945, t044, t223 and t860 which were detected in 27 (6.7%), 23 (5.7%), 19 (4.7%) and 18 (4.5%) isolates, respectively. Spa types t002 and t030 were each detected in 11 (2.7%) isolates; t019 was detected in 10 (2.5%) isolates. The other spa types occurred sporadically. A population snap shot of the relationship of the spa types is presented in Fig 1. It divided the isolates into eight spa clonal complexes (Spa-CC) with Spa-CC421 as dominant followed by Spa-CC223.

Twenty-seven sequence types (STs) were identified among the 103 selected for MLST. For the remaining 297 isolates, ST was predicted based on their spa types. The majority of the isolates (209; 52.2%) belonged to ST239. This was followed by ST22 (37; 9.2%), ST80 (30; 7.5%), ST5 (N = 26; 6.5%), ST30 (N = 14; 3.5%), ST241 (N = 11; 2.7%), ST6 (N = 9; 2.2%), ST36 (N = 8; 2%), and ST772 (N = 8; 2%). ST8 was detected in seven (1.7%) isolates, while ST97 was detected in six (1.5%) isolates. Four (1%) isolates, each belonged to ST1, ST88, ST121 and ST913. ST113 and ST225 were detected in three (0.7%) isolates each, whereas ST105, ST149 and ST361 were each detected in two (0.5%) isolates. The other sequence types (STs) consisting of ST46, ST72, ST508, ST1289, ST1465, ST1637 occurred in single isolates (0.2%). A new sequence type, ST2816 was described in this study.

### Characterization of MRSA clones in Kuwait hospitals

The application of eBurst identified 13 clonal complexes and one singleton, while the combination of MLST and SCC*mec* typing had identified 32 different MRSA clones. The distribution of MRSA clones and their resistance and virulence genes profiles are shown in Tables 2–5. All of the tested MRSA isolates carried combination of genes encoding virulence factors including hemolysins, leukocidins, Staphylococcal enterotoxins, Staphylococcal superantigen/enterotoxin-like genes (ssl/set), epidermal cell differentiation inhibitors (edinA, edinB, edinC). In addition, they carried at least one of the genes encoding proteases such as aureolysin encoding genes (aur), serine protease (splA, splB, splE), glutamylendopeptidase (sspA), staphopain B protease (sspB), staphopain A protease (sspP). Furthermore, they carried adhesion factors/MSCRAMM encoding genes including bone sialoprotein-binding protein (bbp), collagen-binding adhesion (cna), clumping factors (ClfA, ClfB), cell surface elastin binding protein (epbs), enolase (eno), fibrinogen binding protein (fib), fibronectin-binding protein A (fnbA), S. aureus surface protein G (sasG), Ser-Asp rich fibrinogen-/bone sialoprotein-binding proteins (sdrC, sdrD), van Willebrand factor binding protein (vwb), surface protein involved in biofilm formation (bap), and at least one of the biofilm encoding gene, ica (icaA, icaC, icaD). In addition, nearly all strains carried hyaluronate lyase (hysA).

**Table 2:** Characteristics of CC1, CC5, CC6 MRSA in Kuwait hospitals.

MRSA clones	Spa type	Resistance phenotype	Antibiotic resistance genes	Virulence genes
**CC1**				
ST1-V	t6811	E,CC,FD	*fusC (Q6GD50)*, *ermC*	*sea*, *sec*, *seh*, *sek*, *sel*, *seq*
ST1-V	t127, t321	CN, K, TE, FD, CIP, TOB,	*fusC (Q6GD50)*, *aacA-aphD*, *aphA3*, *sat*	PVL, *sea*, *seh*, *sek*, *seq*, *ebh*
ST772-V	t345, *t10795	K,AK,E,CC,TE,W,CIP,TOB	*msrA*, *mphC*, *aphA3*, *Sat*, *tetK*, *fosB*	PVL, *sea*, *egc*
ST772-V	t657, t12211	CN,K,AK,E,W,CIP,TOB	*msrA*, *mphC*, *aacA-aphD*, *aphA3*, *Sat*, *fosB*	PVL, *sea*, *sec*, *sel*, *egc*
**CC5**				
ST105-II	t002	K,AK,E,CC,FD,SH,CIP,TOB	*ermA*, *msrA*,*mphC*, *aadD*, *aphA3*, *Sat*, *fosB*	TSST, *sea*, *egc*
ST5-II	t105	CN,K,E,CC,W,SH,CIP,TOB	*ermA*,*aacA-aphD*, *dfrS1*, *mupA*, *fosB*	*sed*, *sej*, *ser*, *egc*
ST5-II	t242	E,CC,SH,CIP	*ermA*, *fosB*	*sed*, *sej*, *ser*, *egc*
ST5/ST225-II	t003/t045	K,AK,E,CC,FD,SH,CIP,TOB	*ermA*, *aadD*, *fosB*	*sea*, *sed*, *sej*, *ser*, *egc*
ST5-IV	t306, t2164	E,CC	*ermC*, *fosB*	PVL, *sea*, *sed*, *sej*, *ser*, *egc*
ST149-IV	**t1154, t2164	E,CC,FD	*ermC*, *fusC (Q6GD50)*, *fosB*	*sea*, *egc*
ST5-V	t688	TE	*tetK*, *tetM*, *fexA*, *fosB*	*sea*, *sed*, *sej*, *ser*, *egc*, LukF-PV (P83)
ST1637-V	t5258	CN,K,AK,TOB	*aacA-aphD*, *fosB*	*sea*, *sed*, *sej*, *ser*, *egc*
ST5-VI	t688	C,TE,W,RF,FD	*XylR*,*Vga*,*dfrA*,*tetM*, *fexA*,*fosB*	*sed*, *sej*, *ser*, *egc*, LukF-PV (P83)
**CC6**				
ST6-IV	t304	-	*fosB*	*sea*
ST6-IV	t304	E,CC	*ermC*, *fosB*	-

*the isolate also carried sed.

**the isolate also carried TSST, sec and sel.

**Abbreviations:** Ak, amikacin; C, chloramphenicol; CN, gentamicin; CIP, ciprofloxacin; CC, clindamycin; E, erythromycin; FD, fusidic acid; K, kanamycin; HLR-Mup, high-level mupirocin resistance; RF, rifampicin; TE, tetracycline; TOB, tobramycin; SH, spectinomycin; W, trimethoprim.

**Table 3:** Characteristics of CC8 MRSA in Kuwait hospitals.

MRSA clones	Spa type	Resistance phenotype	Antibiotic resistance genes	Virulence genes
**CC8**				
ST8-IV	t008	K,E,CIP	*msrA*, *mphC aphA3*, *Sat*, *fosB*	PVL, *sek*, *seq*, ACME
ST8-IV	t008	K,E,CC,SH,CIP,TOB	*ermA*, *aadD*, *qacA*, *fosB*, *merA*, *merB*	*sea*
ST8-IV	^**a**^t064	CN,K,AK,E,CC,TE,W, CIP,TOB, HLR-MUP	*ermC*, *aacA-aphD*, *aadD*, *qacC*, *dfrS1*, *mupA*,*tetM*, *fosB*	*sea*, *seb*, *sek*, *seq*,LukF-PV (P83)
ST113-IV	t064	CN,K,E,CC,C,TE,W,CIP,TOB	*ermC*, *aacA-aphD*, *tetK*, *cat*,*fosB*	*sea*, *seb*, *sed*, *sek*, *seq*,LukF-PV (P83)
ST113-IV	t064	CN,K,E,CC,W,CIP,TOB	*ermC*, *aacA-aphD*, *aphA3*, *Sat*,*fosB*	-
ST8-V	t064	CN,K,E,CC,CIP,TOB	*ermC*, *aacA-aphD*, *fosB*	*sea*, *seb*, *sek*, *seq*
ST239-III	t421	CN,K,AK,E,CC,TE,FD,SH,CIP,TOB	*ermA*, *aacA-aphD*, *aphA3*,*qacA*, *Sat*, *tetK*, *tetM*, *fosB*,*merA*,*merB*	*sea*, *sek*, *seq*
ST239-III	t030	CN,K,AK,E,CC,TE,RF,SH,CIP,TOB	*ermA*, *ermC*, *aacA-aphD*, *tetM*, *fosB*	*sea*, *sek*, *seq* ACME
^**b**^ST239-III	^**c**^t037,t421,t860,t945	CN,K,AK,E,CC,TE,W,FD, TOB	*ermA*,*aacA-aphD*, *tetM*, *fosB*,	*sek*, *seq*
^**d**^ST421-III	t037, t138, t1902	CN,K,AK,E,CC,TE,W,FD,TOB	*ermA*, *aacA-aphD*, *aphA3*, *tetM*, *fosB*, *merA*, *merB*	*sek*, *seq*
ST1465-III	t459	CN,K,AK,E,CC,TE,RF,SH,CIP,TOB	*ermA*, *ermC*, *aacA-aphD*,*tetM*,*fosB*	*sea*,*sed*,*sek*, *seq*,ACME

^a^One of the isolates obtained in 2001 lacked LukF-PV (P83).

^b^ST239-III isolates carried *sea*, *sak*, *chp*, *scn*, *ermC*, *lnuA aphA3*, *qacA*, *qacC*, *Sat*, *tetK*, *cat*, *merA*, *merB*, *mupA* variably.

^c^One t037 isolate obtained in 2005 lacked *ermA*.

^d^ST241-III isolates carried *fnbB*, *ermC*, *tetK*, *qacA*, *qacC*, *sat*, *cat* variably.

**Table 4:** Characteristics of CC22, CC30, CC45, ST72, CC80 MRSA in Kuwait hospitals.

MRSA clones	Spa type	Resistance phenotype	Antibiotic resistance genes	Virulence genes
**CC22**				
ST22-IV	^**a**^t032, ^**b**^t790	E,CC,CIP	*ermC*	*egc*
ST22-IV	t223 (2005)	E,CC,TE,W,	*ermC*, *tetK*, *dfrS1*	TSST, *egc*
ST22-IV	t223 (2010), t5708	TE, W	*tetK*, *dfrS1*	TSST, *egc*
ST22-IV	t223 (2010), t309, ^**c**^t3935	W	*dfrS1*	TSST, *egc*
ST22-IV	t2251	TE	*tetK*	TSST, *egc*
ST22-IV	t852	CN,K,W,CIP,TOB	*aacA-aphD*,*aadD*	PVL, *egc*
ST22-IV	t3107, t5983	CN,K,E,CC,W	*ermC*, *aacA-aphD*,*aadD*, *dfrS1*	PVL, *egc*
**CC30**				
ST30-IV	t019, ^**d**^t345	-	*fosB*	PVL, *egc*
ST30-IV	*t318, t1130	E,CC,SH,W	*ermA*, *dfrS1*, *fosB*	PVL, *egc*
ST36-II	**t018	K,E,CC	*ermA*, *aadD*, *fosB*	*sea*, *egc*
ST36-II	t605	CN,K,E,CC,TE,W,SH,CIP,TOB	*ermA*, *aacA-aphD*, *aadD*, *qacC*, *dfrS1*, *tetK*, *fosB*	TSST, sea, egc
**CC45**				
ST46-IV	t370	K	*aphA3*, *Sat*	*egc*
ST508-IV	t050	-	*-*	*seb*, *egc*, ACME
**ST72**				
ST72-V	t4000	CN,K,TE,FD,TOB	*aacA-aphD*, *fusC (Q6GD50)*, *tetK*, *fosB*	*sec*, *sel*, *egc*
**CC80**				
ST80-IV	***t376, t8154	E, CC	*ermC*	*etD*
ST80-IV	t1154, t018, **§**t042, **¥**t044	K,TE,FD	*aphA3*, *Sat*, *far1*, *tetK*	PVL, *etD*

^**a**^ One of the isolates obtained in 2005 also carried *seb*, *sec*, and *sel*.

^**b**^ the isolate also carried sec.

^**c**^ the isolate also carried *sea*.

^**d**^ the isolate also carried *sea*.

*t318 isolate was resistant to trimethoprim but lacked *dfrS1* and lacked chp-encoding gene.

**t018 isolate obtained in 1999 carried *aphA3*, *sat*, *tetK*, TSST but lacked sea, while the isolate obtained in 2010 carried *ermC*, *aacA-aphD*, *dfrS1*, and *qacC*.

***the isolate carried *seb*, *sek*, *seq*.

**§**t042 isolate lacked *tetK*.

**¥** one of the t044 isolates obtained in 2001 lacked *tetK*, while the isolate obtained in 2010 carried *qacC*.

**Table 5:** Characteristics of CC88, CC97, CC121, CC361, ST2816 MRSA in Kuwait hospitals.

MRSA clones	Spa type	Resistance phenotype	Antibiotic resistance genes	Virulence genes
**CC88**				
ST88-IV	*t690, **t4067, t5041	E, CC, TE	*ermC*, *tetK*	PVL, *sea*
ST1289	t5562	W	*dfrS1*	-
**CC97**				
ST97-V	t1234, t13204	-	*-*	Sak, scn
**CC121**				
ST121-IV	t314	W	*dfrS1*, *fosB*	PVL, *seb*, *egc*
ST121-V	t314	-	*fosB*	PVL, *seb*, *egc*
**CC361**				
ST361-IV	***t315, ****t1427	K	*aphA3*, *Sat*, *fosB*	TSST, *sel*, *egc*
**CC913**				
ST913-IV	t991	-	*ermC*	*Sed*, *etA*, *etD*
**New sequence type**				
ST2816-V	t605	TE, CIP	*tetK*	-

*the t690 isolate carried *sek*, *seq* and LukF-PV and lacked LukS-PV.

** The t4067 isolate was sensitive to tetracycline and lacked tet gene.

*** The t315 isolate also carried *sec*.

****the t1427 isolate also carried *seb*.

#### Clonal complex 1 (CC1)

The seven isolates that belonged to CC1 were obtained in 2010. They consisted of two sequence types; ST1-V (three isolates) and ST772-V (four isolates). The ST1-V isolates corresponds to the ST1-MRSA-V *SCCfus* clone, while the ST772-V isolates correspond to the Bengal Bay/WA MRSA-60 clone. The ST1-V isolates consisted of three spa types, t127, t321, and t6811, which were positive for *agrIII* and *cap8* but varied in their carriage of PVL and antibiotic resistance genes. ST1-V-t6811 isolate was negative for PVL but resistant to erythromycin and clindamycin mediated by *ermC*, whereas t127 and t321 isolates were positive for PVL, and were multiresistant to gentamicin, kanamycin, tetracycline, fusidic acid, ciprofloxacin, tobramycin and carried *aacA-aphD*, *aphA3*, *fusC*, *sdrM* and *sat* determinants.

The ST772-V isolates corresponds to the Bengal Bay/WA MRSA-60 clone. The ST772-V isolates belonged to four spa types, t345, t657, t10795 and a novel spa type t12211 described during this study. All ST772-V isolates were positive for *agrII*, *cap5*, *sea*, *egc* gene cluster (*seg*, *sei*, *selm*, *seln*, *selo*, *selu*) and PVL but differed in the carriage of *sec* and *sel* (Table 2). All isolates were multiresistant and carried *msrA*, *mphC*, *aphA3*, *tetK*, *sat*, *fosB* and *sdrM*. In addition, ST772-V-t657/t12211 isolates were resistant to gentamicin and carried *aacA-aphD* (Table 2).

#### Clonal complex 5 (CC5)

The CC5 isolates were detected from 2001 to -2010. They belonged to six clones consisting of **(i)** ST105-MRSA-II-t002 [tst+] isolates which corresponds to the New York-Japan clone, **(ii)** the ST5-II-003/t105/t242 and ST225-II-t045 [tst-] isolate which correspond to the New York-Japan/Rhine-Hesse EMRSA clone, **(iii)** The ST5-IV-t306/t2164 isolates which correspond to the Pediatric clone, **(iv)** The ST149-IV-t1154/t2164 (*fusC*) isolates which corresponds to the Maltese clone, **(v)** ST5-V-t688 and ST1637-V-t5258 isolates carrying *sed*, *sej*, *ser* which corresponds to WA-MRSA-11/34/35/90/108, and (**vi)** the ST5-VI-t688 isolate which corresponds to the New Pediatric clone.

All CC5 isolates harbored *agrII*, and *cap*5 and the genes for virulence determinants and antibiotic resistance summarized in Table 2.

ST105-II-t002 isolate was PVL-negative, but was positive for tst gene in addition to genes, *sea* and *egc* gene cluster.

The ST5-II-003/t105/t242 and ST225-II-t045 isolates were PVL- and *tst*-negative but carried enterotoxins genes *sed*, *sej*, *ser*, *egc* as shown in Table 2. In addition, t003 and t045 isolates carried an additional gene, *sea*. The ST5-II-t003/t105/t242 and ST225-II-t045 isolates were resistant to erythromycin and clindamycin, and carried *ermA*. However, each spa type showed differences in the carriage of *aacA-aphD*, *aadD*, *dfrS1*, and *mupA* (Table 2).

The ST5-IV clone consisted of two spa types, t306 and t2164, which were PVL-positive and carried similar enterotoxins genes, *sea*, *sed*, *sej*, *ser* and *egc*, and contained *ermC* mediated erythromycin and clindamycin resistance (Table 2). The ST149-IV was PVL-negative but carried *sea* and *egc*. In addition, ST149-IV-t1154 isolate was positive for *sec*, *sel* and *tst*. All ST149-IV-t1154/t2164 isolates were resistant to erythromycin, clindamycin and fusidic acid and *carried ermC*, *fusC*, *fosB* and *sdrM*.

The ST5-V-t688 isolate carried *sea*, *sed*, *sej*, *ser* and *egc* and was sensitive to the majority of the antibiotics tested in this study except tetracycline mediated by *tetK* and *tetM*. In addition, the isolate harbored *fexA*, *fosB* and *sdrM* determinants. The ST1637-V-t5258 isolate was PVL-negative, but was positive for *sea*, *sed*, *sej*, *ser* and *egc* and was resistant to the aminoglycosides, gentamicin, kanamycin, amikacin and tobramycin and harbored *aacA-aphD*, *fosB* and *sdrM* determinants.

The ST5-VI-t688 clone harbored *sed*, *sej*, *ser* and *egc* and were multiresistant carrying *XylR*, *vga*, *dfrA*, *tetM*, *fexA*, *fosB* and *sdrM*.

#### Clonal complex 6 (CC6)

The two representative CC6 isolates selected for microarray analysis carried *sea*, *agrI*, *cap8* and the virulence and antibiotic resistance genes summarized in Table 2. Both ST6-IV-t304 isolates resembled WA MRSA-51. Although isolated in 2005 and 2010, they had identical virulence genes profile but different resistance genes. The 2005 isolate was resistant only to cefoxitin, penicillin, and harbored *sdrM* and *fosB* determinants, whereas the 2010 isolate, was resistant to erythromycin, clindamycin and fusidic acid and carried *ermC*, *sdrM* and *fosB* determinants.

#### Clonal complex 8 (CC8)

Thirty-seven of the 110 isolates selected for microarray analysis belonged to CC8. The CC8 isolates belonged to six STs and 10 spa types. The STs consisted of ST239- III (19 isolates), ST241- III (10 isolates), ST8- IV (four isolates), ST113-IV (two isolates), ST8-V (one isolate), and ST1465-III (one isolate). All CC8 isolates were positive for *agrI* and *cap8* but carried relatively few enterotoxins encoding genes (Table 3).

Microarray analysis classified the CC8 isolates into six established clones. These were: **(i)** ST239-III-t030/t037/t421/t860/t945, ST241-III-t037/t138/t1902 and ST1465-III-t459 isolates; **(ii)** ST8-IV-t064 and ST113-IV-t064; **(iii)** ST8-IV-t008 PVL/ACME-negative isolate; **(iv)** the PVL-positive -ACME-positive ST8-IV-t008, **(v)** ST113-IV-t064; **(vi)** CC8-MRSA-V consisted of ST8-V-t064 isolate.

The majority of the CC8 isolates consisted of ST239-III, ST241-III and ST1465-III and resembled the Brazilian/Hungarian/Vienna clone. All CC8 isolates belonged to *agr1* and *cap8* except one isolate ST239-III-t421 obtained in 1997 yielded negative results for the four known agr types. The ST239/ST241/ST1465-III isolates were multiply resistant to antibiotics as shown in Table 3 and carried similar virulence genes except for *sea* and ACME. Isolates that were positive for *sea* were also positive for ACME. One isolate obtained in 1997 expressed high-level resistance to mupirocin and carried *mupA* (Table 3). All ST239/ST241/ST1465-III isolates were resistant to erythromycin and carried *ermA*. However, five isolates obtained in 1999, 2002 and 2010 carried *ermC* and one isolate obtained in 2005 lacked *ermA* and *ermC*. The ST239-III-t037 isolates, except three isolates obtained in 1999, 2005 and 2010 harbored quaternary ammonium compound resistance genes, *qacA* or *qacC*. Two isolates obtained in 1996 and 2010 were resistant to chloramphenicol and carried *cat* (Table 3).

The ST8-IV-t064 (two isolates) and ST113-IV-t064 (one isolate) clones that were isolated in 2001–2002 and 2010, respectively, resembled USA500 clone. All three isolates were PVL- and ACME-negative but carried enterotoxin genes, *sea*, *seb*, *sek* and *seq*. ST113-IV-t064 carried an additional enterotoxin gene, *sed*. All three isolates were resistant to gentamicin, kanamycin, tobramycin, erythromycin, clindamycin, tetracycline, trimethoprim and ciprofloxacin and carried *ermC*, *aacA-aphD* and *fosB*. Although, both clones were resistant to tetracycline, ST8-IV-t064 isolates carried *tetM*, while ST113-IV-t064 isolate carried *tetK*. ST8-IV-t064 and ST113-IV-t064 isolates differed in their resistance to amikacin, chloramphenicol and high-level mupirocin. Whereas, ST8-IV-t064 isolates were resistant to amikacin and high-level mupirocin resistance and carried *aadD*, *mupA* and *qacC*, the ST113-IV-t064 isolate was resistant to chloramphenicol and carried cat gene. One isolate belonging to ST8-IV-t064 was resistant to teicoplanin (MIC: 24 μg/L) but lacked *vanZ*.

The ST8-IV-t008 isolate, resembling the Lyon/UK EMRSA-2 clone was isolated in 2005. It was negative for PVL and ACME but positive for *sea*.

The ST8-IV-t008 [PVL+] [ACME+] that resembled the USA300 was isolated in 2010. The isolate carried enterotoxin genes, *sek* and *seq*.

ST113-IV-t064 isolate that resembled UK EMRSA-14/WA MRSA-5 clone obtained in 2005 was negative for PVL, ACME and enterotoxin encoding genes.

The ST8-V-t064 isolate yielded negative results for PVL and ACME but carried *sea*, *seb*, *sek* and *seq*.

#### Clonal complex 22 (CC22)

CC22 consisted of 37 isolates, all of which belonged to ST22. Thirteen isolates obtained in 2005 (three isolates) and 2010 (10 isolates) were selected for microarray analysis. The ST22 isolates belonged to 10 spa types including t032, t223, t309, t790, t852, t2251, t3107, t3935, t5708 and t5983. All representative ST22 isolates were positive for *agrI* and *cap5*. The ST22-IV isolates were positive for virulence genes and antibiotic resistance genes summarized in Table 4.

Microarray analysis classified the ST22-IV isolates into three groups comprising (i) t032-SCC*mec* IVh and t790-SCC*mec* IVa obtained in 2005 and 2010, respectively, were related to UK EMRSA-15/Barnim-EMRSA clone; (ii) the t223 (2005 and 2010), t309 (2010), t2251 (2005), t3935 (2010) and t5708 (2010) isolates were related to UK EMRSA-15/Middle Eastern variant; and (iii) the t852, t3107 and t5983 isolates obtained in 2010 were related to CC22-MRSA-IV [PVL+] clone.

#### Clonal complex 30 (CC30)

Isolates belonging to CC30 were first isolated in 1996 and continued to be isolated until 2010. The 11 CC30 isolates selected for microarray analysis belonged to two sequence types, ST30-IV (seven isolates) and ST36-II (four isolates). The ST30-IV isolates belonged to spa types, t019, t318, t345 and t1130, whereas the ST36-II isolates belonged to spa types t018 and t605. Microarray analysis revealed that the ST30-IV isolates were similar to the CC30-IV [PVL+] Southwest Pacific clone, whereas the ST36-II isolates were similar to the UK EMRSA-16 clone.

The ST30-IV-t019/t318/t345/t1130 isolates which resembled the Southwest Pacific clone were positive for PVL, *agrIII*, *cap8* and *egc*. The ST30-IV-t345 carried an additional enterotoxin gene, *sea*. The ST30 isolates expressed different antibiotic resistance determinants. While the ST30-IV-t1130 isolate expressed resistance to erythromycin, clindamycin, spectinomycin and trimethoprim and harbored *ermA*, *sdrM*, *dfrS1* and *fosB*, the ST30-IV-t019/t345 isolates were susceptible to the non-beta-lactam antibiotics tested but harbored *sdrM* and *fosB*. The ST30-IV-t318 isolate was also resistant to erythromycin, clindamycin and spectinomycin and carried *ermA* (Table 4).

The ST36-II isolates consisted of ST36-II-t018 (N = 3) and ST36-II-t605 (N = 1). All ST36-II were positive for *agrIII*, *cap8* and *egc* gene cluster. All isolates were PVL-negative. Two isolates obtained in 1999 (t018 and t605) were *tst*-positive. The ST36-II isolates were resistant to kanamycin, erythromycin, clindamycin, spectinomycin and ciprofloxacin, and carried *ermA*, *aadD*, *sdrM* and *fosB*. Of the three ST36-II-t018 isolates, one isolate obtained in 2010 was resistant to gentamicin and carried additional *aacA-aphD* and additional erythromycin-resistance determinant, *ermC*. Another isolate obtained in 1999 carried *aphA3*. Two isolates obtained in 1999 were resistant to tetracycline and carried *tetK*, while two isolates obtained in 1999 and 2010 were resistant to trimethoprim and carried *dfrS1* (Table 4).

#### Clonal complex 45 (CC45)

CC45 was represented by two isolates; ST46-IV-t370 and ST508-IV-t050 detected in 2001–2002 and 2005 respectively. Microarray analysis revealed that clones ST46-IV-t370 resembled the Berlin EMRSA clone, whereas ST508-IV-t050 resembled the CC45-MRSA-IV [ACME+]. Both clones were positive for *agrI* and *cap*8. The ST46-IV-t370 isolate was PVL and ACME-negative and carried *egc* and *aphA3* encoding kanamycin resistance (Table 4). The ST508-IV-t050 isolate was PVL-negative but was positive for ACME, *seb* and *egc*, and was sensitive to all non-beta lactams antibiotics (Table 4).

#### Sequence types 72 (ST72)

One isolate, ST72-V-t4000 which resembled WA MRSA-91 was negative for PVL and ACME but was positive for *sec*, *sel* and *egc*. The isolate was resistant to gentamicin, kanamycin, tetracycline, tobramycin and fusidic acid and carried *aacA-aphD*, *tetkK* and *fusc* (Table 4).

#### Clonal complex 80 (CC80)

Isolates belonging to ST80-IV were obtained in 1997 (one isolate), 1998 (one isolate), 1999 (two isolate), 2001 (three isolate), 2005 (one isolate) and 2010 (four isolate). The isolates belonged to six spa types including t018, t042, t044, t376, t1154, t8154. However, the majority (seven isolates) of the isolates obtained in 1997, 1998, 1999, 2001, 2005 and 2010 belonged to t044. All the ST80-IV representative isolates were positive for *agrIII* and *cap*8.

The PVL-positive ST80-IV isolates were similar to the CC80-IV [PVL+] European CA-MRSA clone, while the PVL-negative ST80-IV isolates defined a different CC80-MRSA variant. Genes for virulence and antibiotic resistance of CC80 are summarized in Table 4.

#### Clonal complex 88 (CC88)

Four CC88 representative isolates, consisting of ST88 (three isolates) and ST1289 (one isolate) were selected for microarray analysis. The three ST88 isolates were ST88-IV-t690, ST88- IV-t4067 and ST88-IV-t5041. The ST1289-IV isolate belonged to spa type t5562. All CC88 isolates were positive for *agrIII* and *cap8*. Based on microarray analysis, the [PVL^+^] ST88-V isolate correspond to the CC88-MRSA-IV [PVL^+^] group, while the [PVL^-^] ST1289-IV resembled WA MRSA-2 (Table 5).

The ST88-IV isolates differed in their carriage of enterotoxin encoding genes. The ST88-IV-t4067/t5041 isolates carried *sea*, while the ST88-IV-t690 isolate carried *sea*, *sek* and *seq*. One t690 isolate carried lukF-PV and lacked lukS-PV. The isolates were also non-multiresistant to antibiotics and carried few antibiotic resistance genes. All the ST88-IV isolates were resistant to erythromycin and clindamycin and carried *ermC*. In addition, the two ST88-IV isolates belonging to t690 and t5041 were resistant to tetracycline and carried *tetK*. The ST1289-IV-t5562 (WA MRSA-2 clone) isolate lacked enterotoxin and PVL genes, and was only resistant to trimethoprim and carried *dfrS1* (Table 5).

#### Clonal complex 97 (CC97)

All four CC97 isolates were obtained in 2010. The isolates belonged to ST97-V and spa types t1234 and t13204. All CC97 isolates were positive for *agrI* and *cap5*,*sak*, *scn*, but were negative for genes encoding staphylococcal enterotoxins, PVL, TSST-1, ET and ACME (Table 5). The CC97 isolates were susceptible to non-beta-lactam antibiotics tested, although they carried *sdrM* (Table 5).

#### Clonal complex 121 (CC121)

The three representatives of CC121 isolates belonged to ST121-IV-t314 (one isolates) and ST121-V-t314 (two isolates) obtained in 2005 and 2010, respectively. The ST121 isolates were positive for PVL, *seb*, egc, *agrIV* and *cap8* (Table 5). The isolates were sensitive to most of the antibiotics tested but harbored *fosB* and *sdrM*. One ST121-IV-t314 isolate was resistant to trimethoprim and carried *dfrS1* (Table 5).

#### Clonal complex 361 (CC361)

CC361 consisted of two isolates, ST361-IV-t315 and ST361-IV-t1427, that were obtained in 2010. Microarray analysis showed that ST361-IV-t315/t1427 isolates resembled WA MRSA-29. The isolates were positive for *tst*, *agrI*, *cap*8, *sel* and *egc* but differed in their carriage of *seb* and *sec*. The ST361-IV-t1427 isolate carried *seb*, while the ST361-IV-t315 isolate carried *sec* determinant (Table 5). The ST361-IV isolates were resistant to kanamycin and carried *aphA3*. All isolates carried *sat*, *fosB* and *sdrM* (Table 5).

#### Clonal complex (CC913)

CC913 isolates were all isolated in 2010 and were represented by four isolates that belonged to ST913-IV-t991. A representative isolate selected for microarray analysis was positive for *agrII*, *cap8*, *sed*, *etA*, *etD* and the resistance determinants *ermC* and *sdrM* (Table 5).

#### New sequence type (ST)

A new sequence type, ST2816, was identified during this study. The isolate carried SCC*mec*-V and belonged to spa type t605. The isolate was negative for enterotoxins, PVL, TSST-1, ET and ACME but was positive for *ebh*, *fnbB*, *map*, *sak* and *scn* (Table 5). It was resistant to tetracycline and ciprofloxacin and carried *tetk*.

### Changes in the prevalence of MRSA clones from 1992 to 2010

Table 6 summarizes the changes in the numbers and types of MRSA clones from 1992 to 2010. The results showed that the number of MRSA clones changed from one major clone in 1992 to 30 different clones in 2010.

**Table 6:** Distribution of MRSA clones among MRSA isolates in 1992–2010.

Year	No. of MRSA clones	*HA-MRSA clones (ᵃN)	*CA-MRSA clones (ᵃN)
1992	1	ST239-III-t037 (15)	-
1996	3	ST239-III-t037/t421 (17)	ST30-IV-t019 (2)
		ST241-III t1902 (1)	
1997	3	ST239-III-t421 (18)	ST80-IV-t044 (1)
		ST241-III-t037 (13)	
1998	3	ST241-III-t037 (5)	ST80-IV-t044 (1)
		ST239-III-t037/t421 (10)	
1999	5	ST36-II-t018/t605 (3)	ST30-IV-t019 (2)
		ST239-III-t037/t421 (9)	ST80-IV-t044/ t1154 (2)
		ST241-III-t037 (5)	
2001–2002	8	ST5-II-t105 (1)	ST5-IV-t306 (1)
		ST239-III-t421 (14)	ST8-IV-t064 (2)
		ST241-III-t037/t138 (12)	ST30-IV-t318 (2)
			ST80-IV-t044/t018/t852 (6)
			ST508-IV-t050 (1)
2005	11	ST36-II-t018 (2)	ST6-IV-t304 (1)
		ST22-IV-t032/t223/t2251 (4)	ST8-IV-t008 (1)
		ST239-III-t037/t421/t945 (32)	ST30-IV-t019/t345 (2)
			ST46-IV-t370 (1)
			ST72-V-t4000 (1)
			ST80-IV-t044 (5)
			ST113-IV-t064 (2)
			ST121-V-t314 (1)
2010	30		
	ᵇ(12)	ST22-IV-t032/t223/t852/t309/t3935/t3107/t790/t5708/t5983 (33)	ST6-IV-t304 (8)
		ST36-II-t018 (3)	ST5-IV-t002/t688/t2164 (10)
		ST239-III-t030/t037/t421/t860/t945 (69)	ST8-IV-t008 (1)
		ST241-III-t037 (1)	ST30-IV-t019/t345/t1130 (7)
		ST5-II-t003/t242 (2)	ST80-IV-t044/ t042/t376/t8154 (14)
			ST113-IV-t064 (1)
			ST121-V-t314 (1)
	ᶜ(18)	ST105-II-t002 (4)	ST1-V-t127/t321/t6811 (4)
		ST225-II-t045 (3)	ST5-V-t688/2164 (9)
		ST1465-III-t459 (1)	ST5-VI-t688 (1)
			ST8-IV-t008 (USA300) (1)
			ST8-V-t064 (2)
			ST88-IV-t690/t5041/t4067 (4)
			ST97-V-t1234/ t13204 (6)
			ST121-IV-t314 (2)
			ST149-IV- t1154/t2164 (2)
			ST361-IV-t315/t1427 (2)
			ST772-V- t345/t657/t10795/t12211 (7)
			ST913-IV-t991 (4)
			ST1289-IV-t5562 (1)
			ST1637-V-t5258 (1)
			ST2816-V-t605 (1)

ᵃNo. of MRSA isolates.

ᵇMRSA clones detected in the 90’s and the early 2000’s.

ᶜMRSA clones detected only in 2010.

*MRSA strains were classified as HA-MRSA or CA-MRSA based on the carriage of SCCmec types. HA-MRSA carries SCCmec I, II and III+ST22-IV. CA-MRSA carry SCCmec IV (except ST22), V, VI and others [5, 61].

The 15 representative isolates obtained in 1992 belonged to the same clone, ST239-III-t037. However, the number of clones increased to three in 1996 and five in 1999. In 1996, three clones, ST239-III-t037/t421, ST30-IV-t019 and ST241-III-t1902 were detected. Therefore, 1996 marked the introduction of the ST30-IV, the Southwest Pacific CA-MRSA clone, into Kuwait hospitals. Similarly, 1997 marked the introduction of ST80-IV, a European CA-MRSA clone. The types and numbers of MRSA clones detected in 1998 remained the same as the previous years; but by 1999, the number of MRSA clones increased to five and marked the introduction of ST36-II, the Epidemic-MRSA-16 (EMRSA-16) clone.

The number of MRSA clones increased to eight among isolates obtained in 2001–2002. The new clones that appeared during this period included ST8-IV-t064, ST5-IV-t306, ST5-II-t105 and ST508-IV-t050. Eleven clones were identified in 2005 and included seven new clones, ST22-IV-t032/t223/ t2251, ST113-IV-t064, ST6-IV-t304, ST8-IV-t008, ST46-IV-t370, ST72-V-t4000 and ST121-V-t314.

Thirty MRSA clones were detected among the 205 representative MRSA isolates obtained in 2010. These consisted of 12 clones that were identified in previous years and 18 new clones (Table 6). Additionally, it was observed that clones detected in previous years were associated with different spa types in 2010. For example, the ST5-IV clone previously detected in 2001–2002 belonged to spa type t306, but ST5-IV that were isolated in 2010, were associated with spa types, t002, t688 and t2164. Similarly, ST5-II clone that was detected in 2001–2002 was associated with spa type t105, but in 2010, the ST5-II was detected in four isolates that were associated with spa types, t002, t003 and t242. Similarly, the 33 ST22 isolates obtained in 2010 contained new spa types, t852, t790, t3107, t309, t3935, t5708 and t5983 in addition to spa types, t223 and t032 that were detected in 2005.

The ST239-III-t037/t421/t945 clone was found in 69 isolates in 2010 including spa type, t860 that was not detected in previous years.

The 18 new clones that appeared in 2010 consisted of three HA-MRSA clones, ST225-II-t045, ST105-II-t002, and ST1465-III-t459, and 15 CA-MRSA clones consisting ST5-V-t688/t2164, ST772-V-t345/t657/t10795/t12211, ST97-V-t1234/t13204, ST1-V-t127/t321/t6811, ST88-IV-t690/t4067/t5041, ST913-IV-t991, ST8-V-t064, ST121-IV-t314, ST149-IV-t1154/t2164, ST361-IV-t315/t1427, USA300/ST8-IV-t008, ST5-VI-t688, ST1289-IV-t5562, ST1637-V-t5258 and ST2816-V-t605.

## Discussion

This study revealed a striking diversity in the numbers and types of MRSA clones obtained in Kuwait hospitals from 1992 to 2010. Although, several MRSA clones were identified, the majority (64.2%) corresponded to the well-established healthcare-associated ST239-III and ST22-IV MRSA clones [5].

Microarray analysis distinguished two types of ST239-III isolates in this study. One type, consisting of spa types, t037/t421/t860/t945, resembled the well characterized pandemic clone also known as the Hungarian/Brazilian clone [8, 9] which is one of the most successful MRSA lineages reported in many parts of the world [29, 30, 31, 32] including the Middle East [33, 34, 35]. The isolates in this group were positive for *sea*, *sek* and *seq* but lacked PVL and ACME. These were similar to ST239 strains reported in Saudi Arabia [33], Turkey [34], Russia [36] and Germany [37]. The second type consisted of ST239-III-t030 and ST1465-III-t459, characterized by their carriage of ACME genes. ACME has been widely reported among ST8-IV (USA300) [38] clone but less commonly in other MRSA clones [39, 40]. However, ACME-positive ST239-III MRSA isolates have been reported in Australia [10], Singapore [41], Malaysia [42] and strains belonging to other genetic backgrounds such as ST764-SCC*mec*-IIa obtained from cases of acute otitis media in Japan [43] indicating that ACME is spreading among MRSA with different genetic backgrounds. This report of ACME in MRSA isolates obtained in Kuwait hospitals signifies the emergence of ACME producing MRSA clone in our region.

Microarray analysis of antibiotic resistance genes in these isolates yielded two interesting results. First, it showed that none of the fusidic acid resistant ST239-MRSA-III isolates carried *fusB* or *fusC* determinants included in the microarray panel suggesting the involvement of an additional mechanism of fusidic acid resistance such as *fusA* [44]. A previous study in Kuwait showed that fusidic acid resistant MRSA isolates expressed chromosomally-mediated high levels of fusidic acid (MIC: ≥ 256 μg/L) suggesting the presence of *fusA* [45]. Therefore, the isolates in this study may harbor *fusA*. Secondly, the microarray results revealed the presence of *fosB* encoded fosfomycin resistance that was not previously reported in MRSA isolates in Kuwait. Fosfomycin susceptibility testing is not performed routinely in Kuwait, which explains why the resistance was missed even though a substantial number of the isolates were resistant. The detection of this resistance by microarray highlights the benefits of this technology over traditional antibiotic susceptibility testing in detecting novel resistance determinants.

ST22-IV clone was the second most common clone isolated in Kuwait hospitals. It is a well-known epidemic MRSA clone that emerged in the United Kingdom in the early 1990s [13] and has become prevalent in Europe since then [31, 46, 47, 48]. The results of this study showed that ST22-IV MRSA corresponded to three groups including CC22-UK EMRSA-15/Barnim EMRSA, CC22-MRSA-IV-PVL+ and CC22-tst+ UK EMRSA-15/Middle Eastern variant clones. Interestingly, the majority of the isolates were related to the tst+ UK EMRSA-15/Middle Eastern variant [15]. The diversity of the ST22-IV variants circulating in Kuwait hospitals may reflect diverse sources of their acquisition.

The other major MRSA clones detected during this study included ST80-IV, ST30-IV, ST6-IV and ST772-V. ST80-IV- MRSA was previously reported as the most common CA-MRSA clone in Kuwait hospitals in 2005–2006 [16, 17] and was postulated to consist of European and some indigenous clones due to differences in their antibiotic resistance phenotypes [17]. The present results showed that the ST80-IV MRSA were heterogeneous, belonged to different spa types including t044, t042, t8154 and t376 which confirms previous suggestions of their diverse route of acquisitions [17]. Surprisingly only 2.9% (6/205) of the isolates obtained in 2010 were ST30-IV-MRSA. Whereas in our previous studies ST30-IV-MRSA was the second dominant CA-MRSA in Kuwait hospitals with prevalence of 30% and 22% of MRSA isolated in 2001–2003 and 2006 respectively [16,17]. The reduced number of ST30-IVMRSA obtained in 2010 may reflect changes in the MRSA population in Kuwait hospitals.

ST6-IV-t304 MRSA was recently reported as the dominant MRSA clone in a hospital in Oman [35]. Spa type t304 has also been reported in MRSA isolates in UAE [49], MSSA obtained from patients in Lebanon [50] and in MSSA from Camels in UAE [5] suggesting that the strains may be transmitted between humans and Camels. It would be interesting to undertake further investigations into the prevalence of this clone in the region.

The ST772-V MRSA, known as the Bengal Bay clone because of its origin in Bangladesh and India [51], is becoming the dominant MRSA clone in India [52] but has also been reported in Australia where it is called WA60 [53] and in the United Kingdom [54]. Although ST772-V clone was reported previously from patients in Saudi Arabia [33], UAE [49], Qatar [55] and Oman [35], this is the first report in Kuwait hospitals. ST772 isolates were usually associated with spa type t657. However, other spa types including t10795, t345 and t12211 were detected in small numbers in this study signaling the evolution of variants of the Bengal Bay clone.

The ST8-IV-t008, also known as USA300, was also detected for the first time in Kuwait hospitals. The USA300 is a multiresistant, PVL^+^ CA-MRSA clone that has been recognized as the leading cause of community-associated as well as healthcare-associated infections in North American hospitals [56, 57, 58]. The USA300 MRSA clone is also increasingly being reported in hospitals outside North America [34, 43, 59]. The detection of the USA300 CA-MRSA clone in Kuwait highlights its growing importance as an international epidemic MRSA clone.

The ST5 MRSA isolates were heterogeneous in the carriage of SCC*mec* elements. ST5 isolates carried SCC*mec* II (1.2%), SCC*mec* IV (2.7%), SCC*mec* V (2.2%) and SCC*mec* VI (0.2%). Isolates carrying SCC*mec* II genetic element are classified as HA-MRSA whereas those carrying SCC*mec* IV, V and VI are classified as CA-MRSA. Therefore, the ST5 isolates in this study consisted of HA MRSA and CA-MRSA clones. The low prevalence of ST5-IV MRSA in this study (2.7%) mirrors the situation in Asian countries such as Japan, China, Taiwan [60] and Sri Lanka [32] where ST5-IV-MRSA have been rare. Other members of CC5 identified in this study included the ST5/ST1637-V, which was related to WA MRSA 11/34/35/90/108 [53], the ST5-II, related to the New York/Japan MRSA clone, ST149-IV, related to the Maltese clone, and the ST5-VI, which was related to the New Pediatric clone. All of the CC5 isolates carried the *egc* gene cluster and all CC5 isolates, except those related to the Maltese (ST149-IV) and [tst+] New York/Japan clones (ST5-II) carried an additional enterotoxin, *ser* [61]. The egc gene cluster was also common in CC5 isolates reported from Western Australia [53], Malta [61], Germany [37], Ireland [62] and USA [63]. In addition, this study and studies from Malta and Ireland reported the presence of *sea* and *sej* among the CC5 isolates [61,62]. Whereas, ST5-IV (Pediatric clone) were PVL-positive, *tst* was found in ST105-II (New York/Japan clone) and ST149-IV (Maltese clone) isolates. ST97-V isolates were detected in six MRSA isolates in this study. ST97-MRSA-V isolates were previously reported to cause an outbreak in a neonatal unit of a Kuwait hospital in 2007 [64]. However, the ST97-V isolates obtained in 2010 in this study were all susceptible to non-beta-lactam antibiotics; whereas the 2007 outbreak isolates were resistant to gentamicin, kanamycin and fusidic acid indicating that the two ST97-V isolates were unrelated. The ST97 isolates carried sak and scn encoding genes (immune evasion cluster/IEC type E) suggesting that they are of human origin [7].

In addition to revealing the diversity of MRSA clones that circulated in Kuwait hospitals from 1992 to 2010, the study also demonstrated changes in the composition of the MRSA clones during the same period. The distribution of the MRSA clones in Kuwait hospitals remained largely unchanged from 1992 to 1999, as the clonal composition of MRSA was mostly those of healthcare-acquired MRSA represented by ST239-III, the Brazilian/Hungarian MRSA clone. Despite the increase in the number of MRSA clones from 1996 onwards, the ST239-III clone continued to be the dominant clone in Kuwait hospitals. Similar to the result of this study, ST239-III has remained the predominant MRSA clone in Saudi Arabia [33] and Malaysia [42]. Other studies have reported the replacement of ST239-III MRSA as the dominant MRSA clone by CA-MRSA clones in UAE [49], Singapore [65] and Portugal [66], India, ST239-III was replaced by CC22-MRSA-IV and ST772-MRSA-V [52]. Similarly, displacement of ST239-III MRSA by ST22-MRSA-IV has been reported in Germany [67] and Czech Republic [47]. These results highlight the differences in the distribution of MRSA clones in different countries.

The emergence of CA-MRSA in the mid 90's changed the epidemiology of MRSA globally [68]. The present study has shown that CA-MRSA appeared in Kuwait hospitals in 1996 with the detection of ST30-MRSA-IV, the Southwest Pacific clone (SWP), followed by ST80-IV in 1997. The other CA-MRSA clones appeared in 2000 and beyond. It is interesting that the ST30-IV MRSA isolates appeared in Kuwait hospital in 1996 a year after it was reported in New Zealand [69]. It is possible that a South West Pacific national or a returning Kuwaiti tourist to that region introduced it to Kuwait.

The prevalence of ST80-IV, the European CA-MRSA clone, increased in from its introduction in 1997 until 2010. Curiously, ST80-IV-MRSA has not been reported as extensively in Asian countries as in European countries. A surveillance conducted by Song *et al*., [32], in Asian countries including Korea, Taiwan, Hong Kong, Thailand, Vietnam, India, Sri Lanka and the Philippines) did not detect ST80-IV-MRSA, and only few studies in Malaysia [70] and Singapore [71] reported the presence of ST80 in small numbers of their MRSA isolates. In contrast, ST80-IV has become the most common CA-MRSA clones in the Middle East and North Africa [16, 17, 33, 49, 72, 73]. The presence of ST80 isolates in the Middle East and not in other parts of Asia highlights the yet unexplained observations whereby certain clones flourish in certain places but not in others.

The number of MRSA clones increased from one in 1992 to 30 in 2010 including 18 new ones. The 18 new clones that appeared in 2010 included three HA-MRSA clones, ST105-II, ST225-II, ST1465-III, and 15 CA-MRSA clones consisting of USA300, ST1-V, ST5-V, ST5-VI, ST8-V, ST88-IV, ST97-V, ST121-IV, ST149-IV, ST361-IV, ST772-V, ST913-IV, ST1289-IV, ST1637-V and ST2816-V, highlighting the growth of CA-MRSA isolates in 2010. Two of these clones, ST772-V and ST8-IV-t008 [PVL+] (USA300) are recognized epidemic MRSA clones. Although ST239-III was the dominant clone, it decreased in prevalence from 59.6% in 2005 to 33.6% in 2010 as the prevalence of diverse CA-MRSA clones increased.

In conclusion, the results of this study has shown that MRSA isolated in Kuwait hospitals belonged to diverse genetic backgrounds suggesting multiple routes of their acquisition. The majority of the isolates were related to the established healthcare-associated MRSA clones, ST239-III and ST22-IV. However, spa typing revealed that the isolates were heterogeneous representing imported and local clones. The study also revealed a shift in the clonal composition of MRSA isolates in Kuwait hospitals overtime. The number of MRSA clones increased from one in 1992 to 30 in 2010. In the 90’s and early 2000’s, the majority of the isolates belonged to ST239-III. Although ST239-III remained the dominant clone in 2010, other clones have appeared with the majority identified as CA-MRSA.
